# Characteristics of Allergic Pulmonary Inflammation in CXCR3Knockout Mice Sensitized and Challenged with House Dust Mite Protein

**DOI:** 10.1371/journal.pone.0162905

**Published:** 2016-10-11

**Authors:** Zhongjuan Liu, Huaxia Chen, Xiaolan Chen, Jinming Gao, Zijian Guo

**Affiliations:** 1 Department of Laboratory Medicine, Peking Union Medical College Hospital, Chinese Academy of Medical Sciences, Beijing, China; 2 Department of Respiratory Diseases, Peking Union Medical College Hospital, Chinese Academy of Medical Sciences, Beijing, China; University of Michigan Health System, UNITED STATES

## Abstract

Chemokine C-X-C motif receptor 3 (CXCR3) is a chemokine receptor that is mainly expressed by activated T lymphocytes. T cells play important roles in allergic pulmonary inflammation, which is a hallmark of asthma and elicits the localized accumulation of activated T cells in the lung. In China, a marked increase in the incidence rate of chronic allergic pulmonary inflammation has made it a major public health threat. In the present study, we investigated the role of CXCR3 and its ligands in airway inflammation induced by house dust mite protein (HDMP) in a CXCR3 knockout (CXCR3KO) asthma mouse model. Pathological manifestations in the lung, cell counts and bronchoalveolar lavage fluid (BALF) classifications were studied using hematoxylin and eosin (H&E) staining. The levels of IL-4 and IFN-γ in the BALF and splenocyte supernatants were measured using ELISA. CD4^+^ and CD8^+^ T cells in the lung and spleen were analyzed by flow cytometry. RT-PCR was applied to measure the mRNA transcript levels of monokines induced by IFN-γ(CXCL9) and IFN-γ inducible protein 10(CXCL10). The total cell counts, eosinophil counts, and IL-4 levels in the BALF and cultured splenocyte supernatants were significantly increased, while the levels of IFN-γ were reduced in the HDMP groups(*P*<0.01). Changes in the total cell counts, eosinophil counts, and lymphocyte counts, as well as the total protein levels in the BALF, the levels of IL-4 in splenocyte supernatants, and the pathological manifestations in the lung, were all greater in CXCR3KO mice than in C57BL/6 wild-type mice. Furthermore, the expression levels of CXCL9 and CXCL10 mRNA transcripts in the lungs of CXCR3KO mice were lower than those in C57BL/6 wild-type mice (*P*<0.05). CXCR3 and its ligands (i.e., CXCL9 and CXCL10) may play anti-inflammatory roles in this animal model. Promoting the expression of CXCR3 and its ligands may represent a novel therapeutic approach for preventing and curing asthma.

## Introduction

Asthma is a type of airway inflammatory cell infiltration, obstruction and hyper-responsiveness that is characteristic of chronic allergic inflammation [1,2]. Currently, there are approximately 300 million asthma patients worldwide. Approximately 10% of these patients have severe, uncontrolled asthma [3,4]. The onset of asthma is a very complex process; previous studies have identified many mediators that contribute to the development of this disease.

One key step in the initiation of asthma is the persistent recruitment of inflammatory cells into the airways in response to allergens, such as air pollution particles, pollens and certain food components. House dust mites was one of the most prevalent allergen associated with asthma, and they have been identified throughout the world [5,6]. They are a causal agent of asthma, as their inhalation provokes an exaggerated T helper type II (Th2)-polarized immune response. While the mechanisms underlying allergic sensitization and airway inflammation after exposure to house dust mites have been extensively studied, many questions remain unanswered. Notably, dust mites are the most common allergens for patients with asthma in China. Importantly, the animal model of allergic airway inflammation induced by dust mites recapitulates many of the characteristics that affect asthmatics [7].

Several studies have shown that a T helper type I(Th1) and Th2 cytokine imbalance is a key factor associated with airway inflammation in asthma, together with many inflammatory cells, cytokines, chemokines, and their receptors [8–10]. The Th2 cytokine IL-4 can induce eosinophils to infiltrate sites of inflammation and can promote both IgE synthesis and hypersensitivity, whereas the Th1 cytokine IFN-γ can inhibit eosinophil infiltration and IgE secretion [11,12].

Previous reports have suggested that CXCR3 and its ligands play important roles in Th1 cell responses, inflammatory cell chemotaxis, airway epithelial repair after injury, and other activities [13–15]. CXCR3 is preferentially expressed by activated T cells and eosinophils [16]. One CXCR3 ligand, CXCL10, which is expressed on lung epithelial cells, is a chemoattractant that can recruit activated T cells and eosinophils into inflamed sites [17,18]. Clinical research has indicated that the number of CXCR3^+^ T cells in blood sampled from asthmatic patients was elevated, and this elevation was associated with asthma severity [19]. In addition, the interaction of CXCL10 with CXCR3 has been reported to contribute to mast cells migration into airway smooth muscle in asthma [20]. In contrast, other reports have indicated that CXCR3 antagonism did not prevent allergen-induced airway hyper-responsiveness or airway inflammation in a mouse allergy model, despite activity exhibited in vitro [21].

Lin et al. from our laboratory previously found that CXCR3 attenuated the airway hyper-responsiveness and inflammation induced by ovalbumin (OVA) in an animal model [22]. However, there have been no experiments investigating whether the role of CXCR3 is similar in analogous animal model using house dust mite protein (HDMP) instead of OVA. This study aimed to assess the specific effects of CXCR3 in a model of HDMP-induced asthma in CXCR3 knockout (CXCR3KO) mice. Our findings provide novel potential research directions for the treatment of asthma.

## Materials and Methods

### Animals and Materials

Cohorts of 6- to8-week-old SPF CXCR3KO (n = 12, 6 mice each in the experimental and control groups) and C57BL/6 mice (n = 12, 6 mice each in the experimental and control groups) were used. The mice weighed approximately 20±2g.

CXCR3KO mice were produced in the Perlmutter Laboratory at Harvard University using gene-targeting technology and were kindly provided by Dr. Gerard [23]. Wild-type 6 to 8-week-old female SPF C57BL/6 mice were provided by the experimental animal center of the Basic Medicine Research Institute, Chinese Academy of Medical Sciences.

The mice were bred and maintained in a pathogen-free mouse facility at the Peking Union Medical College Animal Care Center, SYXK (Beijing) 2010–0028. Clean food and water were available to the mice *ab libitum*. The mental state, diet and activity of the mice were observed daily. If the animals are found to be less activity, no appetite, no clean hair, soft stool and so on, they were isolated and feed in other cages. These animals are no longer used in experimental studies after checking reason and treating symptomatically. Prior to the experiments, the mice had no disease symptoms. Gender-, age- and weight-matched mice were used in all experiments.

The following 4 groups were used in these experiments: the wild-type mouse control group (A: wild-type control group, WTC); the CXCR3KO mouse control group (B: CXCR3KO control group, KOC); the wild-type mouse HDMP test group (C: wild-type HDMP group, WTP); and the CXCR3KO mouse HDMP test group (D: CXCR3KO HDMP group, KOP).

### Reagents

HDMP (4 g/l) was obtained from the Allergen Research Center of Peking Union Medical College Hospital (Beijing, China). RPMI1640 was purchased from Gibco BRL (Grand island, NY, USA). IL-4 and IFN-γ enzyme-linked immunosorbent assay (ELISA) kits were purchased from R&D Systems (Minneapolis, MN, USA). The following flow cytometry detection reagents were used: phycoerythrin(PE)-labeled CD4 antibody, fluorescein(FITC)-labeled CD8 antibody, and FITC-labeled CD3 antibody all from BD Biosciences (Frabkin, NJ, USA). TRIzol reagent and RT-PCR kits were obtained from Invitrogen (Hudson, CA, USA).

### Instruments

The following instruments were used for the experiments: a Bio-Rad 680 Microplate Reader(CA, USA), a BD Biosciences FACS Calibur Cell Sorting System (Frabkin, NJ, USA), a Beckman Coulter AU-5400 automatic biochemical analyzer(Brea, CA, USA), a 6–1 electrophoresis system(Beijing, China), a PE 9600 PCR instrument (Waltham, MA, USA), and a FR-980A biological electrophoresis image analysis system(Shanghai, China).

### Animal model

On days 1, 3, 5, 7, 9 and 11, mice in the WTP(n = 6) and KOP groups (n = 6) were intraperitoneally injected with 50 μg of HDMP absorbed by 2.25 mg of alum (Pierce) in 200 μl of sterile saline. The WTC(n = 6) and KOC groups (n = 6) were intraperitoneally injected with the same amount of a 0.9% NaCl solution.

On days 13, 16, 19, 20, and 21, the HDMP test groups were challenged with 1 ml of 4 g/l aerosolized HDMP for 30 min in a chamber using a nebulizer, while mice in the control groups were exposed to the same amount of an aerosolized 0.9% NaCl solution alone in the same manner. The experiments were carried out 2 times, each time with half of the experimental animals. The animals in some groups were different because of accidental death during the course of the challenge experiment. All experiments were performed according to international and institutional guidelines for animal care and were approved by the Peking Union Medical College Hospital Ethics Committee for Animal Experimentation (Certificate No: XHDW-2013-003).

At 24 h after the final aerosol challenge, the mice were anesthetized via intraperitoneal injection of 0.3 ml of 10% urethane and euthanized by cervical dislocation. Then, the experimental samples were collected from the mice as described below.

### Recovery of bronchoalveolar lavage fluid (BALF)

After euthanasia, the tracheas of the mice were cannulated using a 20-gauge catheter. BAL was performed 3 times with total of 1.0 ml of ice-cold PBS (pH 7.4). The BALF was centrifuged at 1,500 rpm for 5 min at 4°C. The supernatants were collected and stored at –70°C until subsequent analysis, when the cells were resuspended in 1 ml of PBS. The number of cells in a given volume was counted. A total of 30μl of the residual liquid was added to cytospin slides, which were centrifuged at 800 rpm for 4 min. After being air-dried and fixed with 95% ethanol for 15 min, the slides were ready for routine H&E staining.

### Collection of pathological lung specimens from mice

After the chest was opened, the right lung was ligated, removed and weighed. Then, the lung was placed in a 2-ml tube and stored in liquid nitrogen until RT-PCR.

Approximately 0.3~0.5ml of 10% neutral formaldehyde fixative was injected into the left lung, which was then removed, inflated to 25 cmH_2_O with 10% formalin, and fixed overnight. On the following day, the lungs were embedded in paraffin and sectioned into 5-μm slices, which were then stained with 1% hematoxylin and eosin (H&E). Pulmonary tissues were observed using a light microscope to assess lymphocyte and eosinophil infiltration and airway epithelial injury. Pathological analyses for each mouse were independently performed by 2 pathologists who were blinded to the genotype.

### Splenocyte isolation and culture

After the mice were sacrificed, the spleens were removed and separated into single-cell suspensions under sterile conditions by first grinding the spleens on 200-mesh steel filters. Then, the cells were collected, and red blood cells were lysed after centrifugation at 1,200 rpm for 5 min. The viability of the harvested cells was confirmed to be at least 95% by trypan blue staining. Cells were resuspended in RPMI1640 medium containing 10% fetal bovine serum and were cultured in an incubator at 37°C with 5% CO_2_ for 2 days. The final cell concentration was 1×10^9^/l. Supernatants from the splenocyte cultures were collected and stored at –70°C for the subsequent IL-4 and IFN-γ measurements.

### Measurements of protein levels in BALF

The total protein content in BALF was assayed using a BCA Protein Assay Kit (Thermo Fisher Scientific, China) according to the manufacturer’s instructions.

### ELISA of IL-4 and IFN-γ levels

The concentrations of IL-4 and IFN-γ in the BALF and culture medium were determined using ELISA kits (R&D Systems) according to the manufacturer’s recommendations.

### Labeling cells isolated from BALF and lung tissue

Lung tissue samples were cut into pieces and placed in tubes with trypsin and collagenase buffer. The tissue samples were digested for 1 hour at 37°C, and the cells were then collected by centrifugation. Splenocytes were directly ground on 200-mesh steel filters. Red blood cells were then removed using alysis solution, and the cell concentration was adjusted to 1×10^7^/ml. A total of 100 μl of cells at a concentration of 1×10^7^/ml were recovered from BALF or tissue samples. Next, 10 μl of blocking buffer was added to the cells, which were then incubated for 15 min on ice. After being washed, the cells were incubated with 20 μl of FITC-conjugated anti-CD4 and PE-conjugated anti-CD8 or control mouse IgG2b for 1h on ice in the dark. Then, the cells were washed with PBS and fixed in PBS containing 1% paraformaldehyde. The cells were either analyzed using a BD Biosciences FACS Calibur Cell Sorting System or were stored at 4°C in a refrigerator for subsequent testing. Flow cytometry analysis of various cell fractions was performed using FlowJo software.

### RNA extraction and RT-PCR analysis

Total RNA was extracted from whole lung tissues using guanidine isothiocyanate and was then reverse-transcribed into cDNA using Omniscript Reverse Transcriptase. A total of 100 mg of cryopreserved tissue was placed in an Eppendorf tube; then, 1 ml of TRIzol reagent was added, and the tissue was rapidly minced and homogenized. After centrifugation, the contents of the tube were processed with chloroform and isopropanol via vortexing, which was followed by 75% ethanol precipitation and dissolution in diethylpyrocarbonate-treated water. RNA purity and concentration were evaluated using an ultraviolet spectrophotometer. Electrophoretic analysis of the RNA clearly showed the 18S and 28S rRNA bands, indicating that the RNA was intact.

PCR was performed using a PE 9600 PCR instrument (PE Applied Biosystems) with 1 μl of cDNA in a 20-μl final reaction volume. The RT-PCR products were electrophoresed in an agarose gel, and the gel was imaged. The resulting band intensities were quantified using Bio-Rad Quantity One software. β-Actin was used as an internal control, and the ratio of experimental band intensity to that of the internal control was used to represent relative gene expression.

The following primers were used:

CXCL9: S: 5ʹ-CTTGGGCATCATCTTCCTG-3ʹ; AS: 5ʹ-TGAACGACGACGACTTTGG-3ʹ;

CXCL10: S: 5ʹ-GTCATTTTCTGCCTCATCC-3ʹ; AS: 5ʹ-GAGCCCTTTTAGACCTTTT-3ʹ; and β-actin: S:5ʹ-CTTCCTTAATGTCACGCACGATTTC-3ʹ; AS: 5ʹ-GTGGGGCGGCCCAGGCACCA-3ʹ.

### Statistical analysis

Statistical analyses were performed using SPSS 15.0(SPSS Inc., Chicago, IL). Continuous variables are reported as the mean± standard deviation. Comparisons between two groups were performed using Student’s *t-*test, and multiple groups were compared using a repeated-measures analysis of variance (ANOVA) with Tukey’s test for multiple comparisons. Differences were considered to be statistically significant at *P*<0.05.

## Results

### Pathological changes in the lungs of mice sensitized by and exposed to HDMP

**Fig 1:**
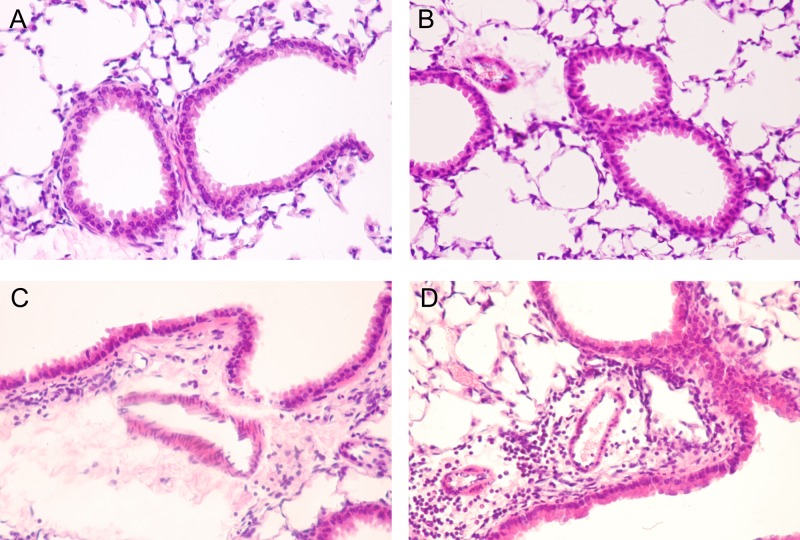
Lung Histopathology (H&E staining). A, WTC group (200×); B, KOC group (200×); C, WTP group (200×); D, KOP group (200×). Mice were sensitized and challenged with HDMP. Lung sections were stained with 1% H&E reagent. Histopathological changes were observed using a light microscope, as described in the Materials and Methods section. A-D, Representative H&E images; n = 6 animals per group.

The bronchi, small vascular submucosa, and surrounding tissues of sensitized CXCR3KO mice showed obvious infiltration by inflammatory cells (mainly eosinophils, lymphocytes, neutrophils, and macrophages; Fig 1D). Many inflammatory cells had migrated into the bronchial and vascular walls, and some of the epithelial cells had been shed. Additionally, a visible mucus plug was present in some bronchi, and the vascular walls showed obvious edema. However, in wild-type mice, inflammatory cell infiltration and eosinophil numbers were significantly lower than those in CXCR3KO mice (Fig 1C). In the WTC and KOC groups, the bronchial epithelium and submucosal layers had remained intact, the bronchial lumen was normal, and no inflammatory cells had infiltrated the surrounding tissues (Fig 1A and 1B).

### Determination of IL-4, IFN-γ, and total protein levels and cytological changes in the BALF

To determine whether CXCR3 depletion affected the antigen-induced infiltration of inflammatory cells into airways, we estimated cell subpopulation sizes in the BALF following antigen sensitization and challenge. Cell changes in the BALF were as follows: the total number of cells and the numbers of lymphocytes, mononuclear macrophages and eosinophils were significantly higher in sensitized mice than in the controls. Compared with sensitized wild-type mice, the presence of various cell types in sensitized CXCR3KO mice was significantly increased, revealing that HDMP sensitization in these mice could induce inflammatory cell infiltration.0020CXCR3KO mice were more likely to show induced inflammation than were wild-type mice (Table 1).

**Table 1:** Changes in Cell Counts (X±S, ×10^4^/ml), IL-4 and INF-γ Levels in BALF (X ± S).

Groups	WTC(n = 4)	WTP(n = 6)	KOC(n = 6)	KOP(n = 5)
Total cells	9.17±1.71	19.79±2.03*	11.54±0.86	31.46±6.15*#
Macrophages	7.98±1.49	15.48±1.58*	10.04±0.75	21.71±3.15*#
Lymphocytes	0.28±0.05	2.38±0.24*	0.35±0.03	4.57±0.43*#
Eosinophils	0.05±0.01	1.76±0.18*	0.06±0.01	3.62±0.44*#
IL-4(pg/ml)	67.11±6.59	114.84±25.22*	68.56±11.15	107.28±20.43*
IFN-γ(pg/ml)	55.60±11.76	34.00±6.83*	57.68±9.26	26.36±7.90*#
Protein(μg/ml)	0.22±0.04	0.21±0.02	0.19±0.04	0.27±0.03*#

WTC, Wild-type control group; KOC, CXCR3KO control group; WTP, wild-type HDMP test group; KOP, CXCR3KO HDMP test group

*P<0.01, HDMP vs. control

#*P*<0.01, CXCR3KO vs. wild-type.

The BALF levels of IL-4 in the WTP and KOP groups were significantly higher than those in the control groups (*P*<0.01). The levels of IFN-γ in the KOP group were significantly reduced compared with the corresponding control group(*P*<0.01). This finding suggests that a Th2-type reaction could induce cytokine production in the lung in response to sensitization and challenge with HDMP in the 2 groups of mice. We also found that the levels of IFN-γ were significantly different between the 2 sensitized groups (Table 1). Additionally, the protein content in the BALF of sensitized CXCR3KO mice was significantly higher than that in the control mice(*P*<0.01). However, there were no significant differences between the sensitized wild-type mice and the corresponding control mice. The protein content in the BALF of sensitized CXCR3KO mice was significantly higher than that in sensitized wild-type mice (*P*<0.01).

### Antigen-specific production of IL-4 and IFN-γ by splenocytes

**Fig 2:**
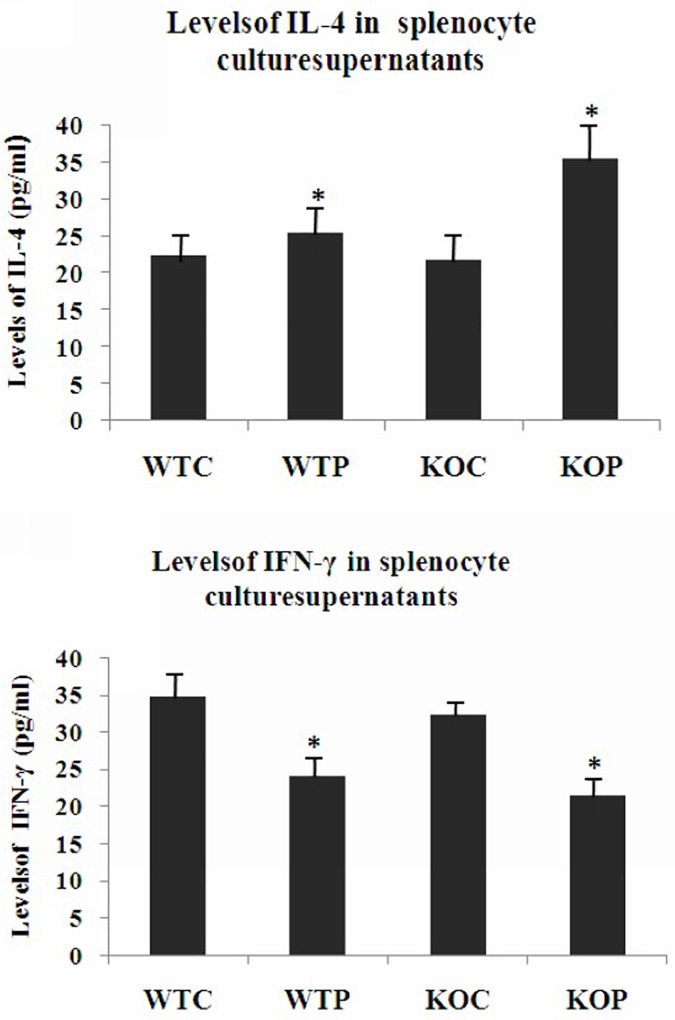
Levels of IL-4 and IFN-γ in Mouse Splenocyte Culture Supernatants. WTC: wild-type control group; KOC: CXCR3KO control group; WTP: wild-type HDMP test group; KOP: CXCR3KO HDMP test group. The levels of IL-4 were significantly increased in splenocyte culture supernatant derived from sensitized mice, and the levels of IFN-γ were decreased. Furthermore, the level of IL-4 was significantly different between the KOP and WTP groups; WTC: n = 4; WTP: n = 6; KOC: n = 6; KOP: n = 5; *P<0.01, HDMP vs. control; #*P*<0.01, CXCR3KO vs. wild-type.

Splenocytes from the sensitized groups of mice were purified and stimulated with HDMP in vitro. We found that the supernatant levels of IL-4 from the splenocytes of sensitized mice were significantly higher than those of the controls(*P*<0.01). Furthermore, the supernatant levels of IL-4 from splenocytes of sensitized CXCR3KO mice were significantly higher than those from sensitized wild-type mice (*P*<0.01). The supernatant levels of IFN-γ from splenocytes in the sensitized group were significantly lower than those in the control group (*P*<0.01). In addition, the supernatant levels of IFN-γ from the splenocytes of sensitized CXCR3KO mice were lower than those from wild-type mice, but the difference was not significant (Fig 2).

### CD4^+^ and CD8^+^ T cells in lung tissue samples

**Fig 3:**
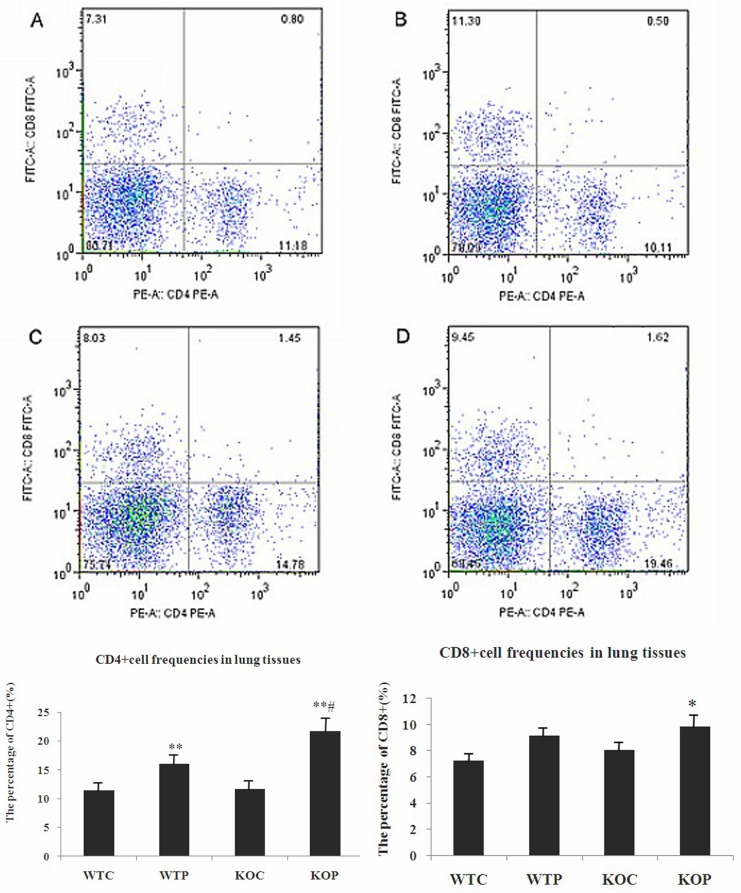
Flow Cytometric Assessment of CD4^+^ and CD8^+^ T Cell Frequencies in the Lung. WTC: wild-type control group; KOC: CXCR3KO control group; WTP: wild-type HDMP test group; KOP: CXCR3KO HDMP test group. Flow cytometry was used to detect T cell subsets in the lung. Top panel, representative histogram showing the presence of CD4^+^ T cells and CD8^+^ T cells in lung tissue. The data presented one of four animals in 2 independent experiments. Bottom panel, pooled data showing the percentages of CD4^+^ T cells and CD8^+^ T cells in lung tissue; n = 4; *P<0.05 and **P<0.01, HDMP vs. control; #*P*<0.01, CXCR3KO vs. wild-type.

Flow cytometry was used to detect T cell subtypes after the mouse lung tissue samples were digested. We found that CD4^+^ T cells in sensitized wild-type mice were significantly more abundant than in non-sensitized mice. Additionally, CD4^+^ T cells in sensitized CXCR3KO mice were also significantly more abundant than in non-sensitized mice *P*<0.01and *P*<0.01, respectively). The proportion of CD4^+^ T cells was also significantly greater in sensitized CXCR3KO mice than in sensitized wild-type mice (*P*<0.01). In addition, the percentage of CD8^+^ T cells was significantly greater in the KOP group than in the KOC group (*P*<0.05). However, the percentages of CD8^+^ T cells in the WTP and the KOP groups were not significantly different (*P*>0.05). The data for Fig 3 are shown in S1 Table.

### CD4^+^and CD8^+^ T cells in the spleen

**Fig 4:**
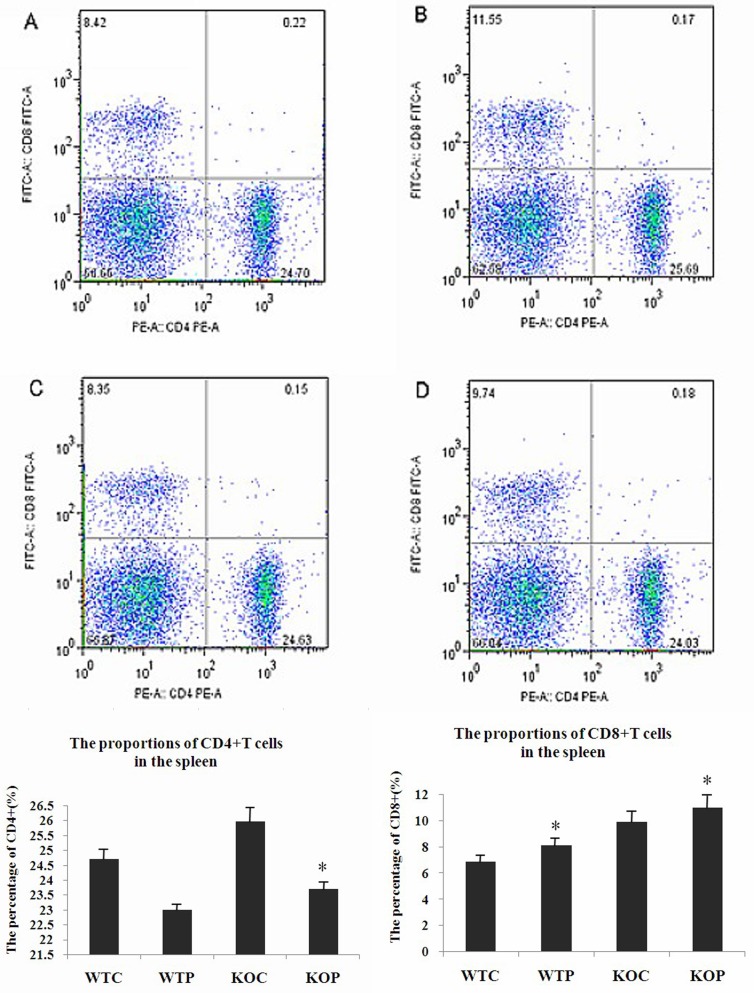
The Proportions of CD4^+^ and CD8^+^ T Cells in the Spleen, as Determined by Flow Cytometry. A, B, C, D represent WTC, KOC, WTP and KOP group respectively. WTC: wild-type control group; KOC: CXCR3KO control group; WTP: wild-type HDMP test group; KOP: CXCR3KO HDMP test group. Top panel, representative histogram showing the presence of CD4^+^ T cells and CD8^+^ T cells in the spleen. The data presented one of four animals in 2 independent experiments. Bottom panel, pooled data showing the percentages of CD4^+^ T cells and CD8^+^ T cells in the spleen; n = 4; *P<0.05.

Flow cytometry was used to detect T cell subsets among isolated splenocytes. Our findings indicated that while the percentage of CD4^+^ T cells in the WTP group was lower than that in the WTC group, the difference was not significant (*P* = 0.08). In the KOP group, the percentage of CD4^+^ T cells was significantly lower than that in the KOC group (*P*<0.05). There were no significant differences between the WTP and KOP groups. CD8^+^ T cells were detected significantly more frequently in the WTP group than in the WTC group (*P*<0.05). There were no significant differences between the KOP and KOC groups or between the WTP and KOP groups. The data for Fig 4 are shown in S2 Table.

### CXCL9 and CXCL10 expression in lung tissue samples

**Fig 5:**
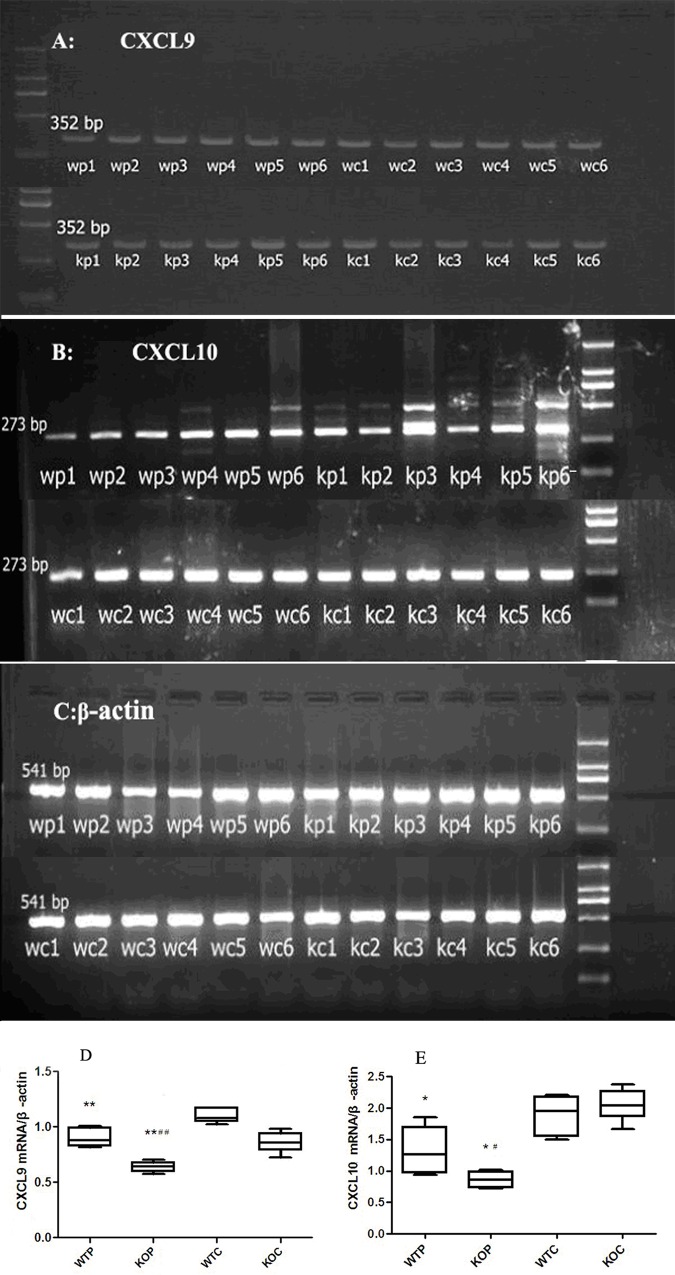
Relative mRNA Transcript Levels of CXCL9 and CXCL10 in Lung Tissue Samples. WTC/WC: wild-type control group; KOC/KC: CXCR3KO control group; WTP/WP: wild-type HDMP test group; KOP/KP: CXCR3KO HDMP test group. The RT-PCR products were electrophoresed on an agarose gel (Fig 5A-5C). Band intensities in the resulting images were quantified using Bio-Rad Quantity One software. β-actin was used as an internal control, and the ratio of the 2 bands was used to represent the relative gene expression value (Fig 5D and 5E); n = 6 mice per group; *P<0.05 and **P<0.01, HDMP vs. control; #*P*<0.05 and ##*P*<0.01, CXCR3KO vs. wild-type.

Total RNA was extracted from lung tissue samples and amplified using RT-PCR. The mRNA transcript levels of CXCL9, CXCL10, and β-actin are shown in Fig 5A–5C; additionally, the relative mRNA transcript levels are shown in Fig 5D–5E. We found that the CXCL9 and CXCL10 mRNA transcript levels in the WTP group were significantly lower than those in the WTC group (*P*<0.01 and *P*<0.05, respectively). The mRNA transcript levels of CXCL9 (Fig 5D) and CXCL10 (Fig 5E) in the KOP group were significantly lower than those in the KOC group (*P*<0.01 and *P*<0.01, respectively). The CXCL9 and CXCL10 mRNA transcript levels in the KOP group were also lower than those in the WTP group (*P*<0.01 and *P*<0.05, respectively).

## Discussion

This study successfully established a mouse model of allergic pulmonary inflammation in wild-type and CXCR3KO mice via an intraperitoneal injection of dust mite protein for sensitization followed by challenge via repeated inhalation of the allergen. In this mouse model, the total number of cells in the BALF increased significantly. The trachea, vascular submucosa and surrounding cells were infiltrated by eosinophils, lymphocytes and macrophages. The lung tissue samples showed pulmonary congestion, edema, and airway epithelial shedding. Some tissues also showed a visible mucus plug [24,25]. All of these observations are typical features of allergic pulmonary inflammation, indicating that the experimental animal model was successfully established.

CXCR3, a Th1-type cell receptor, plays an important role in immune system regulation in asthma. Typically, CXCR3 is selectively expressed on the surface of activated T cells. Its ligands, e.g., CXCL9 and CXCL10, are important for the activation of T cells. Evidence indicates that an imbalance in the differentiation of Th1 and Th2 cells is a key feature of asthma pathogenesis. In asthma, T helper cells in the local microenvironment can differentiate into both Th1 and Th2 cells. Furthermore, these cells can exhibit specific cytokine secretion profiles; for example, IFN-γ indicates a type1 response, and IL-4 represents a type2 response. Before our experiment, we hypothesized that the test results would be similar to those obtained with the OVA-induced asthma model in CXCR3KO mice. Surprisingly, the results were completely different. Two potential explanations for the above results were considered. First, different sensitization methods were used. In the OVA model, sensitization occurred only 2 times, and the mice were challenged 6 times at 10 days after the last injection. In the HDMP model, the mice were sensitized 6 times and were challenged 6 times after the day of the last injection. Second, the mechanism of CXCR3 action may vary among diverse microenvironments [26].

In vivo and in vitro experiments have shown that CXCL9 can reduce the immune responses to challenge with certain antigens in asthma. In a mouse model of asthma, treatment with exogenous CXCL9 significantly reduced airway hyper-responsiveness and eosinophil recruitment [27]. The neutralization of CXCL9 in airways can increase airway hyper-responsiveness and eosinophil recruitment in response to antigen challenge and can also increase IL-4 expression. In vitro, CXCL9 inhibited the chemotaxis of eosinophils in response to eotaxin. CXCL9 and allergen-stimulated lymphocytes can down-regulate IL-4 and up-regulate IFN-γ expression [28]. These findings indicated that CXCL9 can stimulate the conversion of a Th0 responsein to a Th1-polarized response. The main CXCR3 ligand, CXCL10, can significantly enhance Th1 responses to specific antigens in healthy individuals. Campbell et al. [29] found that exogenous ligands for CXCR3 significantly up-regulate IFN-γ secretion by monocytes in peripheral blood from healthy individuals and that this response can be inhibited by an anti-CXCR3 antibody. Furthermore, the levels of CXCR3 ligands in allergic patients were significantly reduced, and IFN-γ levels induced by chemokines did not influence Th2 cells but were closely related to the expression of CXCR3. These findings support a model in which ligands for CXCR3 enhance Th1 cell responses. Low levels of CXCR3 ligands in allergic patients may represent one of the important factors that maintain an allergic response. CXCL9 and CXCL10havealso been reported to be natural CXCR3 receptor antagonists [30]. CXCR3 is a cytokine receptor on the Th2 cell surface and can intracellularly transmit signals, thereby inducing eosinophil aggregation [31]. Therefore, CXCL9 and CXCL10 can antagonize the CXCR3 receptor and inhibit some Th2 cell actions.

In the present study, the total number of cells and the numbers of eosinophils and lymphocytes in the BALF from sensitized CXCR3KO mice were significantly increased compared with those in wild-type mice. The protein content in the BALF of KOP group was significantly higher than that in KOC group and WTP group (*P*<0.01 and *P*<0.01 respectively). The BALF levels of IL-4 in the WTP and KOP groups were significantly higher than those in the control groups (*P*<0.01), and the supernatant levels of IL-4 from splenocytes of sensitized CXCR3KO mice were significantly higher than those from the corresponding control group and sensitized wild-type mice (*P*<0.01).

The inflammatory pathological changes in lung tissues from CXCR3KO mice were also significantly greater than those in wild-type mice, and the tissues showed more eosinophil and lymphocyte infiltration. The IFN-γ levels in the BALF from CXCR3KO mice were lower than in the BALF from wild-type mice. The CXCL9 and CXCL10 mRNA expression levels in lung tissues were also significantly lower in CXCR3KO mice than in wild-type mice. These findings indicate that the allergic pulmonary inflammation was significantly more severe in sensitized CXCR3KO mice than in wild-type mice in response to HDMP.

These findings might be explained by the following model. The chemotaxis of Th1 cells is likely to be reduced in a CXCR3KO context, resulting in fewer Th1 cells involved in the inflammatory response. Moreover, Th1-type cells secrete less IFN-γ, such that the inhibitory effect of IFN-γ on the Th2 cell response is reduced. Furthermore, because IFN-γ secretion is reduced, the chemokines CXCL9, CXCL10 and I-TAC, which are induced by IFN-γ, are also reduced. Thus, CXCL9, CXCL10, and I-TAC become less efficient as enhancers of Th1-type responses and inhibitors of Th2-type responses. Because of the absence of CXCR3 expression, CXCL9, CXCL10, and I-TAC cannot induce downstream signaling via the Th1 cell receptor CXCR3, resulting in further decreases in IFN-γ secretion and the formation of a negative feedback loop.

Our findings suggest that reduced Th1-type cytokine production leads to weaker Th1-type cell responses. Moreover, the reduced levels of CXCL9 and CXCL10 resulted in less Th2 cell inhibition, thereby enhancing Th2 cell responses. Accordingly, the Th1/Th2 cell ratio becomes severely imbalanced, such that CXCR3KO mice show more severe airway inflammation. Our data suggest that the CXCR3 receptor and its ligands enhance Th1 cell responses by this chemokine pathway, which acts to maintain environmental allergen tolerance and may have a potential therapeutic role in rebalancing Th2-type allergic immune responses.

The levels of IL-4 in CXCR3KO splenocyte culture supernatants were significantly increased, whereas those of IFN-γ were significantly reduced. Our findings showed that Th2 cytokines were predominant, which is consistent with the pathological role and features of human allergic asthma.

In conclusion, this study shows that airway inflammatory responses are more severe in CXCR3KO mice than in wild-type mice. This finding might suggest that CXCR3 and its ligands, CXCL9 and CXCL10, may play anti-inflammatory roles in the occurrence and development of asthmatic lung inflammation; thus, they represent protective factors in asthma. Promoting the expression of CXCR3 and its ligands in T cells may represent a novel prevention and treatment strategy for asthma.

## Supporting Information

S1 Table The Proportions of CD4^+^ and CD8^+^ T Cells in the Lungs (n = 4, x±s, %). WTC, KOC, WTP and KOP represent Wild-type control group, CXCR3KO control group, wild-type HDMP test group and CXCR3KO HDMP test group, respectively. *: P<0.01, HDM VS control, #: P<0.01, CXCR3^-/-^ VS wild-type.
(DOCX)

S2 Table The Proportions of CD4^+^ and CD8^+^ T Cells in the Spleen (n = 4, x±s, %). WTC, KOC, WTP and KOP represent Wild-type control group, CXCR3KO control group, wild-type HDMP test group and CXCR3KO HDMP test group, respectively. *: P<0.05, HDM VS control.
(DOCX)
