# Supplementary material for: Characteristics of Allergic Pulmonary Inflammation in CXCR3Knockout Mice Sensitized and Challenged with House Dust Mite Protein
Source: PLoS One. 2016 Oct 11;11(10):e0162905. doi: 10.1371/journal.pone.0162905 (PMC5058494; doi:10.1371/journal.pone.0162905)
Supplement: S1 Table — WTC, KOC, WTP and KOP represent Wild-type control group, CXCR3KO control group, wild-type HDMP test group and CXCR3KO HDMP test group, respectively. *: P<0.01, HDM VS control, #: P<0.01, CXCR3-/- VS wild-type. (DOCX) [file pone.0162905.s001.docx]

**S1 Table. The Proportions of CD4^+^ and CD8^+^ T Cells in the Lungs (n=4, x±s, %)**

| **Groups** | **CD4**^+^ T cells | **CD8**^+^ T cells |
| --- | --- | --- |
| WTC | 11.42±1.17 | 7.22±1.28 |
| KOC | 11.62±0.87 | 8.04±0.73 |
| WTP | 16.06±0.72* | 9.14±1.73 |
| KOP | 21.74±1.21*^#^ | 9.81±1.10* |

WTC, KOC, WTP and KOP represent Wild-type control group, CXCR3KO control group, wild-type HDMP test group and CXCR3KO HDMP test group, respectively.

*: P<0.01, HDM VS control, #: P<0.01, CXCR3^-/-^ VS wild-type
