# Supplementary material for: Characteristics of Allergic Pulmonary Inflammation in CXCR3Knockout Mice Sensitized and Challenged with House Dust Mite Protein
Source: PLoS One. 2016 Oct 11;11(10):e0162905. doi: 10.1371/journal.pone.0162905 (PMC5058494; doi:10.1371/journal.pone.0162905)
Supplement: S2 Table — WTC, KOC, WTP and KOP represent Wild-type control group, CXCR3KO control group, wild-type HDMP test group and CXCR3KO HDMP test group, respectively. *: P<0.05, HDM VS control. (DOCX) [file pone.0162905.s002.docx]

**Table S2 The proportion of CD4^+^ and CD8^+^ T Cells in the Spleen (n=4, x±s, %)**

| **Groups** | **CD4**^+^ T cells | **CD8**^+^ T cells |
| --- | --- | --- |
| WTC | 24.70±0.54 | 6.87±0.69 |
| KOC | 25.96±1.33 | 9.94±0.57 |
| WTP | 23.08±1.62 | 8.11±0.29* |
| KOP | 23.65±0.44* | 9.69±1.16 |

WTC, KOC, WTP and KOP represent Wild-type control group, CXCR3KO control group, wild-type HDMP test group and CXCR3KO HDMP test group, respectively.

*: P<0.05, HDM VS control
